# High-quality production of human α-2,6-sialyltransferase in *Pichia pastoris* requires control over N-terminal truncations by host-inherent protease activities

**DOI:** 10.1186/s12934-014-0138-8

**Published:** 2014-09-11

**Authors:** Doris Ribitsch, Sabine Zitzenbacher, Peter Augustin, Katharina Schmölzer, Tibor Czabany, Christiane Luley-Goedl, Marco Thomann, Christine Jung, Harald Sobek, Rainer Müller, Bernd Nidetzky, Helmut Schwab

**Affiliations:** ACIB - Austrian Centre of Industrial Biotechnology, Petersgasse 14, A-8010 Graz, Austria; Institute of Molecular Biotechnology, Graz University of Technology, Petersgasse 14, A-8010 Graz, Austria; Institute of Biotechnology and Biochemical Engineering, Graz University of Technology, Petersgasse 12, A-8010 Graz, Austria; Roche Diagnostics GmbH, Nonnenwald, D-82377 Penzberg, Germany; Roche Diagnostics GmbH, Pharma Biotech Development, Nonnenwald, D-82377 Penzberg, Germany

**Keywords:** Therapeutic glycoproteins, *In-vitro* glycosylation, Human sialyltransferase, ST6Gal-I, *N*-glycan remodeling, Proteolysis in *Pichia pastoris*

## Abstract

**Background:**

α-2,6-sialyltransferase catalyzes the terminal step of complex *N*-glycan biosynthesis on human glycoproteins, attaching sialic acid to outermost galactosyl residues on otherwise fully assembled branched glycans. This “capping” of *N*-glycans is critical for therapeutic efficacy of pharmaceutical glycoproteins, making the degree of sialylation an important parameter of glycoprotein quality control. Expression of recombinant glycoproteins in mammalian cells usually delivers heterogeneous *N*-glycans, with a minor degree of sialylation. *In-vitro* chemo-enzymatic glycoengineering of the *N*-glycans provides an elegant solution to increase the degree of sialylation for analytical purposes but also possibly for modification of therapeutic proteins.

**Results:**

Human α-2,6-sialyltransferase (ST6Gal-I) was secretory expressed in *P.pastoris* KM71H*.* ST6Gal-I featuring complete deletion of both the N-terminal cytoplasmic tail and the transmembrane domain, and also partial truncation of the stem region up to residue 108 were expressed N-terminally fused to a His or FLAG-Tag. FLAG-tagged proteins proved much more resistant to proteolysis during production than the corresponding His-tagged proteins. Because volumetric transferase activity measured on small-molecule and native glycoprotein acceptor substrates did not correlate to ST6Gal-I in the supernatant, enzymes were purified and characterized in their action on non-sialylated protein-linked and released *N*-glycans, and the respective N-terminal sequences were determined by automated Edman degradation. Irrespective of deletion construct used (Δ27, Δ48, Δ62, Δ89), isolated proteins showed N-terminal processing to a highly similar degree, with prominent truncations at residue 108 - 114, whereby only Δ108ST6Gal-I retained activity. FLAG-tagged Δ108ST6Gal-I was therefore produced and obtained with a yield of 4.5 mg protein/L medium. The protein was isolated and shown by MS to be intact. Purified enzyme exhibited useful activity (0.18 U/mg) for sialylation of different substrates.

**Conclusions:**

Functional expression of human ST6Gal-I as secretory protein in *P.pastoris* necessitates that N-terminal truncations promoted by host-inherent proteases be tightly controlled. N-terminal FLAG-Tag contributes extra stability to the N-terminal region as compared to N-terminal His-Tag. Proteolytic degradation proceeds up to residues 108 – 114 and of the resulting short-form variants, only Δ108ST6Gal-I seems to be active. FLAG-Δ108ST6Gal-I transfers sialic acids to monoclonal antibody substrate with sufficient yields, and because it is stably produced in *P.pastoris*, it is identified here as an interesting glycoengineering catalyst.

**Electronic supplementary material:**

The online version of this article (doi:10.1186/s12934-014-0138-8) contains supplementary material, which is available to authorized users.

## Background

Therapeutic glycoproteins have gained enormous importance in the treatment of serious diseases [1-4]. Clinically used glycoproteins became one of the fastest growing markets in the pharmaceutical industry, accounting for 77 high-value drugs out of 642 pharmaceuticals approved by the European Medicines Agency [4]. One of the most critical quality attributes of these drugs is the N-linked glycosylation which is known to have a dramatic impact on the therapeutic efficacy, serum half-life and immunogenicity [5,6].

Glycosylation is the most complex and widespread posttranslational modification of proteins [7]. The sophisticated glycosylation machinery needed for the biosynthesis of therapeutically acceptable glycoprofiles is most likely provided in mammalian cells [8]. Hence, recombinant therapeutic glycoproteins are mainly produced in Chinese hamster ovary (CHO) cell lines but also in cell lines from baby hamster kidney (BHK-21) and murine tissues [9]. The *N*-glycans of proteins produced in these cells closely resemble the human glycan structure but still differ in crucial points [10-12]. In humans, the terminal monosaccharides of *N*-glycans are partially capped with the sialic acid derivative N-acetylneuramic acid (Neu5Ac) which strongly affects the solubility, stability and immunogenic properties of the respective glycoproteins. Glycoproteins expressed in CHO cells are also incompletely capped at the terminal monosaccharides [13].

To overcome these problems, the *in-vitro* glycosylation of therapeutic proteins by glycosyltransferases (GTs, EC 2.4.) has attracted the interest of the pharmaceutical industry since it offers the opportunity to control the glycosylation of therapeutic proteins to a desired, homogenous and bioactive glycoform [14,15]. *In-vitro* sialylation offers the possibility to complete sialylation of therapeutic glycoproteins for analytical purposes, e.g. for analyzing the effect of sialylation on receptor binding, but also to modify the drug substance itself. Human sialyltransferases are a functional family of at least 20 glycosyltransferases which are subdivided into ST3Gal-, ST6Gal-, ST6GalNAc- and ST8Sia- families [16,17], depending on the acceptor they act on (Gal: galactose, GalNAc: *N*-acetylgalactosamine, Sia: sialic acid) and the linkage they form (α2,3-, α2,6-, α2,8-linkage). The ST6 family includes two subfamilies, ST6Gal-I and ST6Gal-II, both mediating the transfer of sialic acid in α2,6-linkeage from the donor substrate CMP-sialic acid to the terminal galactose but with slight differences in their acceptor specificities [18]. Human ST6Gal-I (β-galactoside α-2,6-sialyltransferase 1, E.C 2.4.99.1) belongs to the CAZy family GT29. Like all mammalian Golgi-resident GTs, ST6Gal-I features a type II transmembrane architecture consisting of an N-cytoplasmic tail, a single transmembrane domain and a large C-terminal catalytic domain facing the luminal side of the Golgi apparatus [19]. The catalytic domain of human ST6Gal-I adopts the predicted global GT-A fold, a seven-stranded central sheet flanked by α-helices, and is stabilized by three disulfide bindings [20]. The domain contains two NxS/T motifs for potential N-glycosylation which is supposed to contribute to the protein stabilization but is not absolutely required for *in-vivo* activity [21].

Much effort has already been expended to express human ST6Gal-I as full-length glycoprotein but without achieving acceptable activities. For instance, ST6Gal-I activity in stably transfected CHO cells was restricted to a crude membrane fraction [22]. ST6Gal-I expressed in *Saccharomyces cerevisiae* was retained in the endoplasmatic rediculum [23] and secretory expression in *Pichia pastoris* resulted in only 10 mU/L culture supernatant [24]. Obviously, the strong hydrophobic character of the transmembrane domain has clearly restrained the translocation, folding and solubility of the enzyme. Consequently, human ST6Gal-I was N-terminally truncated by the hydrophobic structural domains. As a result, an N-terminally truncated ST6Gal was now secretory expressed in *P.pastoris* [25], and transiently expression of truncated ST6Gal-I in HEK293 cells resulted in a considerably improved production rate [20]. In COS cells, truncated ST6Gal-I was secreted with a rate of 10 ng of FLAG-ST6Gal-I/10^6^ cells/h [26]. Expression experiments of ST6Gal-I in CHO cells has shown, that N-terminal truncation of the first 89 amino acids - including the short N-terminal cytoplasmic tail, the transmembrane domain and the stem region - was tolerated even though the acceptor preference got lost, whereas further truncation to residue 100 completely abolished enzymatic activity [27]. The results led to the conclusion that the conserved motif QVWxKDS (aa 94–100 in human ST6Gal-I), which has been found within all sialyltransferase subfamilies, is crucial for activity.

In this work we report on the identification of a minimized catalytic domain of human β-galactoside α-2,6-sialyltransferase 1 corresponding to Δ108ST6Gal-I and its soluble expression in *P.pastoris* for the use in *in-vitro* sialylation of therapeutic proteins. Expression of N-terminally truncated ST6Gal-I variants revealed that the enzyme is proteolytically degraded in *P.pastoris* KM71H. Precise analysis of the degradation products by MS unveiled Δ108ST6Gal-I as the main degradation product. Contrary to the expectations from literature, Δ108ST6Gal-I was found to be active and catalyzed the transfer of sialic acid to a humanized monoclonal antibody IgG1. Variant Δ108ST6Gal-I was successfully expressed in the methylotropic yeast *P.pastoris* in sufficient yields for a potential large scale application.

## Results and discussion

The production of mammalian proteins like sialyltransferases put high requirements on expression systems [28]. Very often expression systems are needed that perform post-translational modifications in order to produce properly folded and active proteins. Hence, eukaryotic expression systems like CHO and BHK cells have been preferably applied for the production of mammalian proteins. However, the production of proteins in mammalian cells is limited due to low expression levels and high production costs. The methylotrophic yeast *Pichia pastoris* offers an alternative expression system since it combines the unique advantages of prokaryotic growth characteristics and high expression levels with the ability to perform posttranslational protein modifications like glycosylation available only in eukaryotic systems [29]. For this reason, *P.pastoris* has been chosen in this work for the functional expression of human ST6Gal-I.

### Protein design for soluble expression of ST6Gal-I

For soluble expression of human ST6Gal-I in *P.pastoris,* the N-terminal hydrophobic topology domains were progressively deleted due to the truncation sites which have been determined from sequence comparison and published truncation experiments [26,27].

**Figure 1:**
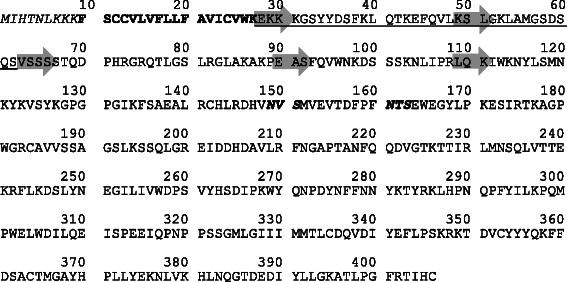
**Structural domains of human ST6Gal-I** (**data base entry P15907).** *Italics*: cytosolic domain (aa 1–9). *Bold*: transmembrane domain (aa 10–27). *Underlined*: stem-region (aa 28–62). *Bold and Italics*: potential N-glycosylation sites. N-termini of truncated variants are indicated by a grey arrow.

According to the literature, the cytoplasmic-, transmembrane- and stem- (CTS) region contributes to the Golgi retention [30]. Analysis of the CTS region by the SACS HMMTOP Algorithm [31] predicts amino acids 1–9 as the NH_2_-terminal cytoplasmic tail (CT) followed by a single transmembrane domain (TMD) ranging from aa 10–27 (Figure 1). The stem region which tethers the catalytic domain is highly variable in length among the human STs and assumed to range from aa 28–60 in human ST6Gal-I. Consequently, the catalytic domain includes amino acids 61–466. Similar boundaries were obtained from isolation of a soluble ST6Gal-I from rat liver tissues lacking a 62 aa NH_2_-terminal peptide which indicates aa 63–466 as the catalytic domain [32]. Altogether, four different variants were constructed by progressive truncation of the structural domains *i.e.* the short NH_2_-terminal cytoplasmic tail and the transmembrane domain (Δ27ST6Gal-I), the stem region (partially and fully, yielding Δ48ST6Gal-I and Δ62ST6Gal-I) and parts of the catalytic domain (Δ89ST6Gal-I). The deletions were located in the hypervariable region outside the conserved domain of human ST6Gal-I [23]. The genes coding for the truncated variants were optimized for expression in *P.pastoris* and fused to an N-terminal 6xHisTag.

### Production in *P.pastoris* KM71H

**Figure 2:**
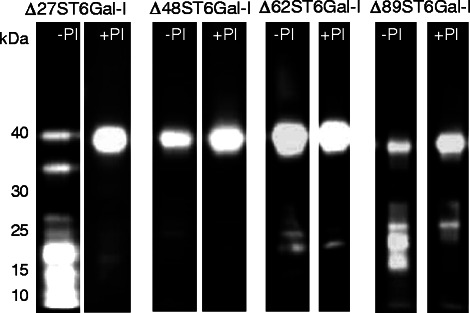
**Expression of N-terminally truncated ST6Gal-I variants in** ***P.pastoris*** **KM71H in the absence (−PI) and presence (+PI) of protease inhibitors.** His-tagged enzyme variants were expressed for 96 h with (+PI) and without (−PI) protease inhibitor. WB analysis of fermentation supernatants (each 600 μL, precipitated with 20% TCA). Immunodetection with HRP conjugated anti-α2,6ST6.

The N-terminally truncated ST6Gal-I variants were produced in *P.pastoris* KM71H as secretory protein. In order to monitor and analyze the expression levels of the ST6Gal-I variants, samples were withdrawn from the cultures at the start of induction (0 h) and 24, 48, 72 and 96 h after addition of MeOH. From each culture, aliquots of fermentation supernatant were precipitated with TCA and analyzed by Western Blot (WB) using two HRP conjugated antibodies for immunodetection, the polyclonal anti-α2,6ST6 and the monoclonal anti-polyHis. The antibodies were selected since they recognized different sequence parts of the protein and enabled the detection of the N-terminus (monoclonal anti-polyHis) as well as the C-terminal sequence (anti-α2,6ST6) of the enzyme. Comparison of samples drawn after 96 h of induction revealed conspicuous protein signals (Figure 2, −PI). In general, strong signals were obtained from detection with anti-α2,6ST6 which indicated that the expression, procession and secretion of ST6Gal-I worked sufficiently in the selected transformants. However, the main protein bands of the variants occurred just below 40 kDa which was lower than the expected molecular weights for the glycosylated enzymes and even lower than the calculated masses for the deglycosylated forms of Δ27ST6Gal-I (44.7 kDa), Δ48ST6Gal-I (42.1 kDa), Δ62ST6Gal-I (40.7 kDa) and Δ89ST6Gal-I (37.9 kDa). Additional protein bands which were observed at very low molecular weights strengthened the presumption that proteolytic degradation has taken place. Moreover, no signals appeared with anti-polyHis (data not shown) which led to the conclusion that the proteolytic degradation proceeded from the N-terminus of each enzyme variant. Generally, the degradation was also detected at shorter induction times (data not shown) and increased with proceeding time. In case of Δ48ST6Gal-I, no degradation products below 40 kDa were detected after 96 h of induction in the presence of protease inhibitor but signals occurred with increasing induction times (data not shown). In order to reduce proteolysis, the expression of variants was repeated with addition of protease inhibitors (Figure 2, +PI). Now, less degradation products were detected with anti-α2,6ST6 but like before, the main protein bands appeared around 40 kDa and no signals were observed with anti-polyHis. Apparently, the addition of protease inhibitors significantly reduced the degradation to smaller products below 20 kDa but could not prevent the first steps of proteolysis concerning the N-terminus of ST6Gal-I including the His-Tag.

After WB analysis, the fermentation supernatants of N-terminal truncated ST6Gal-I variants, expressed in the presence of protease inhibitors, were analyzed regarding their sialyltransferase activity by measuring the transfer of fluorescence-labeled sialic acid from CMP-9-fluoresceinyl-Neu5Ac (CMP-9F-Neu5Ac) to asialofetuin [33]. Measurements at 37°C and pH 6.5 resulted in 34 mU/L for Δ27ST6Gal-I, 80 mU/L for Δ48ST6Gal-I and 115 mU/L for Δ62ST6Gal-I and Δ89ST6Gal-I, respectively. Interestingly, the activities did not correlate with the expression levels of the main protein bands in Figure 2 (+PI) which might indicate that most of the immuno-detected enzyme variants were degraded to less active or inactive products and only small amounts of active protein were existent. In general, the volumetric enzyme activities increased with progressive truncation of ST6Gal-I.

### Functional characterization of ST6Gal-I variants and identification of Δ108ST6Gal-I

Expression analysis of differently truncated ST6Gal-I variants clearly demonstrated that the enzyme was degraded to specific cleavage products accompanied by a loss of activity. For identification of the main cleavage site and its impact on activity, the dominating protein band at approximately 40 kDa of each ST6Gal-I variant (except of the lowest active Δ27ST6Gal-I) was purified from the fermentation supernatant (Additional file 1: Figure S1) and subjected to an extensive characterization. First, the specific activity for the sialic acid transfer from CMP-9F-Neu5Ac to asialofetuin was measured and calculated as RFU/μg. Afterwards, ST6Gal-I was tested regarding sialylation activity using the Fc glycan of a highly galactosylated humanized monoclonal antibody IgG1 as acceptor substrate (Additional file 2: Figure S2). This assay allowed the determination of the sialylation degree, i.e. if one or two sialic acids are attached to the glycan structure. At the start of the sialylation procedure the IgG1 holds no sialic acid residues, just two galactose units (denoted as G2). During incubation with ST6Gal-I, one sialic acid (G2 + 1SA) or even two sialic acids (G2 + 2SA) can get attached to the G2 structure. Determination of G2 + 1SA and G2 + 2SA *N*-glycans was performed by electrospray ionization mass spectrometry of the transfer reaction samples. In a last step, the N-terminal sequence of a purified sample of each variant was determined by Edman degradation. After deglycosylation, the amino acid sequences of the different ST6Gal-I variants were deduced from MS analysis. From each variant, several batches were characterized. The most significant results are summarized in Table 1.

**Table 1:** **Analytic data of N-terminally truncated ST6Gal-I constructs**

**Construct**	**Intensity of variant in ESMS [%]**	**MS analysis**	**Truncation**	**IgG1**		**Asialofetuin**
				**G2 + 1SA [%]**	**G2 + 2SA [%]**	**Fetuin [RFU/** **μg]**
Δ48ST6Gal-I	100	NYLS…	Δ114	7	0	n.d.
Δ62ST6Gal-I*	75	NYLS…	Δ114	22	0	70
	25	WKNYLS…	Δ112			
Δ62ST6Gal-I	30	LQKIWKNYLS…	Δ108	29	0	208
	70	NYLS…	Δ114			
Δ62ST6Gal-I	50	LQKIWKNYLS…	Δ108	51	0	186
	20	NYLS…	Δ114			
	15	IWKNYLS…	Δ111			
	10	WKNYLS…	Δ112			
Δ62ST6Gal-I	100	LQKIWKNYLS…	Δ108	63	34	664
Δ62ST6Gal-I	100	LQKIWKNYLS…	Δ108	82	15	689
Δ89ST6Gal-I	-	-	-	n.d.	n.d.	n.d.

n.d. not detectable.

*Due to the low reproducibility, several batches of variant Δ62ST6Gal-I have been analyzed.

Interestingly, activity of purified Δ48ST6Gal-I towards asialofetuin was not detectable but the variant formed 7% G2 + 1SA when assayed towards IgG1. Variant Δ62ST6Gal-I showed a low batch-to-batch reproducibility which was reflected by fluctuating transfer activities (Table 1). Specific activities ranging from 70 to 689 RFU/μg were obtained for asialofetuin and a similar performance was observed towards IgG1. While batches with lower activities on asialofetuin (70 to 208 RFU/μg) also displayed low capability to sialylate the terminal galactosyl moieties of IgG1 (22 to 29%), batches of Δ62ST6Gal-I which were highly active on asialofetuin (664 and 689 RFU/μg) were able to mask much more of total galactosyl moieties available. Formation of G2 + 1SA was favored (63–82%), however, disialylation (G2 + 2SA) was also observed at a reasonable extent of 15 – 34% (based on initial G2 + 0SA concentration). After purification, variant Δ89ST6Gal-I showed no transfer activity towards asialofetuin or IgG1 anymore. Hence Δ89ST6Gal-I was not further investigated.

Mass analysis of the different constructs confirmed the presumption from WB analysis of the fermentation supernatants with anti-polyHis that in each case the N-terminal sequence of ST6Gal-I has been fully degraded to products corresponding to Δ108 – Δ114 truncations. In general, the transfer activities correlated well with the N-terminal truncations. The low active Δ48ST6Gal-I was found to be solely present as Δ114 product starting with NH_2_-NYLS. Batches of Δ62ST6Gal-I performing best on asialofetuin and IgG1 were strictly existent as Δ108 variants starting with NH_2_-LQKIWKNYLS whereas batches with lower activities on acceptor proteins contained mixtures of truncated products ranging from Δ111 (NH_2_- IWKNYLS) to Δ114 (NH_2_-NYLS). Apparently, the presence of the Δ108ST6Gal-I construct correlated well with the observed transfer activities. Interestingly, Δ108ST6Gal-I was still very active even though significant parts of the predicted catalytic domain were missing. This is contradicting to previous studies [26,27] reporting that the conserved sequence QVWxKDS covering residues 94–100 is crucial for activity. Proceeding degradation from Δ108 to Δ114 drastically reduced and almost abolished enzyme activity as demonstrated for Δ48ST6Gal-I which ended up in 100% of Δ114. Since only low amounts of Δ111 and Δ112 were found we assumed that the degradation from Δ108 to Δ114 proceeds very fast. The results strongly support that not only the stem region but also N-terminal sequence parts of the predicted catalytic domain are highly sensitive to proteolytic processing events like they occur also in nature. Glycosyltransferases are type II membrane proteins and often retained in the Golgi apparatus. However, some of the enzymes are cleaved by endogenous proteases and secreted out of the cell [34]. These proteolytic cleavage events frequently occur in the stem region and release a catalytically active fragment of the glycosyltransferases [34]. Hence, glycosyltransferases have been initially isolated from various body fluids [35]. Soluble ST6Gal-I has been purified first from goat, bovine, and human colostrum [36] and from rat liver missing 63 aa from the N-terminus of the enzyme [32]. Recently, it has been demonstrated that the β-amyloid-converting enzyme 1 (BACE1) is responsible for cleavage and secretion of human ST6Gal-I into the plasma [37]. BACE1 is a membrane-bound aspartic acid protease that cleaves the amyloid precursor protein to produce the neurotoxic amyloid β-peptide, which is a crucial initiation process of the pathogenesis of Alzheimer’s disease. Investigation of this proteolysis by incubation of rat ST6Gal-I (88% similarity to human ST6Gal-I) with BACE1 revealed that the enzyme was first cleaved between Leu^37^ and Gln^38^ to generate the sequence Gln^38^-Ala^39^-*Lys*^40^-*Glu*^41^-Phe^42^ at the N-terminus. In a second step, peptide Gln^38^-Ala^39^-Lys^40^ was trimmed by luminal aminopeptidase(s) yielding Glu^41^ as the N-terminal amino acid of the soluble ST6Gal-I. It is assumed that the generation of soluble ST6Gal-I by BACE1 is needed for the sialylation of soluble plasma glycoproteins to protect them from clearance by the hepatic asialoglycoprotein receptors [37]. This two-step process of proteolytic degradation bears certain resemblance to the degradation of human ST6Gal-I during expression in yeast concerning the way it proceeds. The cleavage started between Arg^108^ and Leu^109^ and proceeded to Asn^115^ in Leu^109^-Gln^110^-Lys^111^-Ile^112^-Trp^113^-*Lys*^114^-*Asn*^115^-Tyr^116^. In both cases the final cleavage takes place after a Lysine residue. The proteolytic sensitivity of the stem region is highly relevant for physiological processes but strongly affects the stability during expression in yeast.

### Expression and characterization of FLAG-Δ108ST6Gal-I

**Figure 3:**
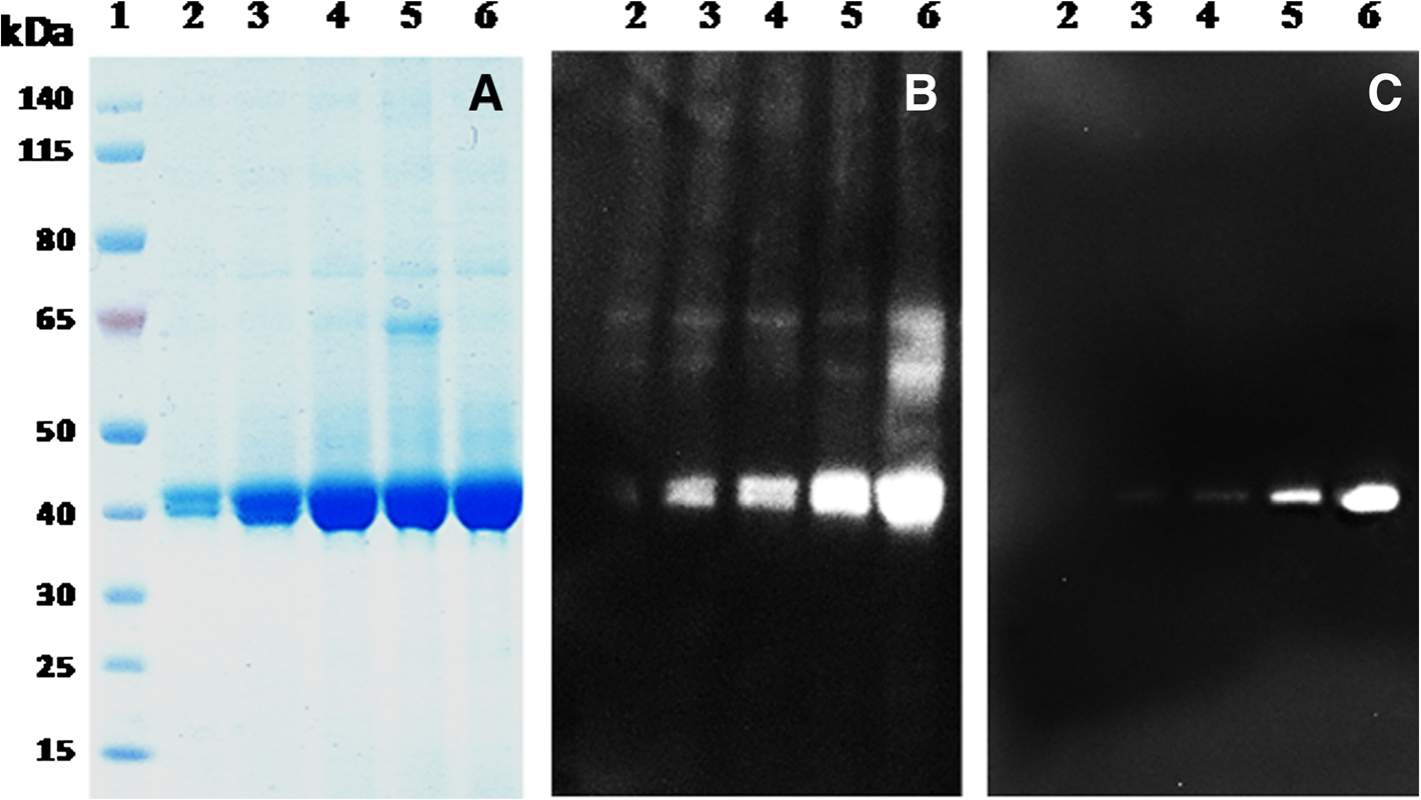
**Expression analysis of human FLAG-Δ108ST6Gal-I in** ***P.pastoris*** **.** Analysis by **A)** SDS-PAGE and Western Blot using **B)** anti-α2,6ST6 and **C)** anti-FLAG for detection. *Lane 1*: Novex sharp protein standard; *lane 2–6*: fermentation supernatant (300 μL TCA precipitated) after 24, 48, 72, 96 and 120 h of induction in shake flasks without addition of protease inhibitor.

In order to confirm the activity of the newly identified Δ108ST6Gal-I variant and to evaluate its potential for large scale application, the variant was cloned into pPICZαB and expressed in *P.pastoris* KM71H without addition of protease inhibitor. In a first approach, variant Δ108ST6Gal-I was expressed without Tag as well as N-terminally fused to the 6xHisTag (Additional file 3: Figure S3). In both cases, strong degradation was detected by Western Blot analysis yielding inactive degradation products as determined by the activity assay. SDS-PAGE analysis of untagged ST6Gal-I revealed high amounts of enzyme at 40 kDa whereas the His-tagged ST6Gal-I was much lower expressed. The results gave reason to suspect that the highly charged HisTag might additionally increase proteolysis. For this reason, the hydrophilic FLAG-Tag peptide was N-terminally fused to the protein for protection against proteolysis yielding FLAG-Δ108ST6Gal-I. SDS-PAGE analysis of culture supernatants drawn at certain time points after induction (Figure 3A) revealed that FLAG-Δ108ST6Gal-I was produced and secreted in unexpected high amounts and purity without overloading the secretory pathway of *P.pastoris.* At all time points, two protein bands, running closely together at about 40 kDa, appeared at the SDS-PAGE. Immunodetection with anti-α2,6ST6 generated two signals (Figure 3B). After stripping, the membrane was re-used for detection with anti-FLAG which recognized solely the upper protein band (Figure 3C). The results indicated that variant FLAG-Δ108ST6Gal-I was not only present as the entire protein but also as an N-terminally truncated variant lacking the FLAG-Tag and presumably a few amino acids of the catalytic domain. The weaker signals obtained with anti-FLAG might probably be caused by the re-usage of the membrane after stripping and do not reflect original protein concentrations. In contrast to the Δ27-Δ89ST6Gal-I variants, no further proteolysis to smaller degradation products did occur, which provides evidence for an improved stability of FLAG-Δ108ST6Gal-I. Due to the highly similar surface charges the two protein bands could not be completely separated by ion exchange chromatography. Hence, a FLAG-Δ108ST6Gal-I variant comprising a certain percentage of truncated and therefore less active or inactive protein was used for further characterization.

So far, only little information has been published on specific activities of ST6Gal-I. This might be attributed to the poor availability of the enzyme. ST6Gal-I isolated from human liver revealed a transfer activity of 1.2 U/mg using CMP-9F-Neu5Ac and asialofetuin as donor and acceptor substrate, respectively [33]. Specific activities of 6 · 10^−5^ U/mg [23] and 3 · 10^−4^ U/mg [38] were obtained for the sialyltransfer from CMP-[^14^C]-Neu5Ac towards asialofetuin for the human full-length ST6Gal-I expressed in *Saccharomyces cerevisiae* before and after up-scale (150-L bioreactor). Expression of a human ST6Gal-I construct, lacking the transmembrane domain, in insect cells yielded an enzyme preparation with 1.6 U/mg towards asialofetuin when assayed with the radio-labeled sugar donor [39]. In this work, the specific activity of FLAG-Δ108ST6Gal-I was not only determined towards high- but also towards low-molecular weight acceptors (Table 2). Using an HPLC-based assay, specific activities of 0.05 and 0.18 U/mg were obtained for asialofetuin and lactosamine, respectively. These values are intermediate to the specific activities reported above. However, comparison with data from literature has to be treated with caution, due to the different assay conditions used and the great variation in enzyme length and glycosylation pattern. But not only transferase activity is an important parameter for enzyme usability in biocatalysis. In general, the main obstacle for application of glycosyltransferases is the presence of different hydrolytic side reactions [40]. Therefore, we also had a look on sialidase activity and donor sugar hydrolysis. While α2,6-sialidase activity was not observed within the detection limit of 10^−5^ · U/mg, CMP-Neu5Ac was hydrolyzed with a specific activity of 0.03 U/mg (Table 2). This hydrolase activity of human ∆108ST6Gal-I is substantial, however, it is still 100-fold lower compared to bacterial sialyltransferases [41,42]. *In-situ* generation of sugar nucleotides could partially compensate for the loss in efficiency of bacterial counterparts [43,44]. Few relevant protein engineering strategies are reported which rely on a more detailed understanding of the underlying molecular mechanism [41]. However this is beyond the scope of this work.

**Table 2:** **Multifunctionality of human FLAG-∆108ST6Gal-I**

**Enzyme activity**	**Donor**	**Acceptor**	**Specific activity [U/mg]**
α2,6-Sialyltransferase	CMP-Neu5Ac	Lactosamine	0.18 ± 0.01
	CMP-Neu5Ac	Asialofetuin	0.05 ± 0.01
CMP-Neu5Ac hydrolase	CMP-Neu5Ac	-	0.03 ± 0.01
α2,6-Sialidase	6′-Sialyllactosamine	-	n.d.

n.d., not detectable.

Expression of a minimized catalytic Δ108 domain in the presence of an N-terminal FLAG-Tag could remarkably improve the stability of ST6Gal-I during enzyme production (Figure 3). Nevertheless, proteolytic degradation did still occur to a certain degree during expression in shake flasks. It is known that temperature, pH and medium composition strongly influence the proteolytic activity of *P.pastoris* [45]. Hence, FLAG-Δ108ST6Gal-I was produced in a 5-L fed-batch bioreactor which offered the opportunity to tightly control the process conditions for potential reduction of proteolysis during the long expression phase. Expression of FLAG-Δ108ST6Gal-I was performed at 20°C and pH 5.2 for 48 h. The culture supernatant was purified *via* anion exchange chromatography. Typically, 4.5 mg of purified protein were obtained from 1 L fermentation supernatant.

Analysis of the N-terminus by Edman degradation (Table 3) confirmed that 85% of the deglycosylated enzyme was present as the entire protein and only low amounts of enzyme were degraded to Δ114 (10%) and Δ115 (5%). In comparison, only 50% of ∆108 but also 50% of ∆114 was detected in shake-flask experiments (data not listed). However, the specific activity (33 RFU/μg) was not significantly increased compared to a value of 20 RFU/μg obtained from shake-flask expression. Summing up, no substantial reduction in proteolysis during expression could be achieved under the fed-batch conditions used. Moreover, FLAG-Δ108ST6Gal-I did only form 14% of the G2 + 1SA *N*-glycan when assayed towards IgG1 and no G2 + 2SA *N*-glycan product was obtained at all. This is an interesting finding because variant Δ62ST6Gal-I, which was fully degraded to Δ108 (Table 1), showed 60–80% G2 + 1SA and 15–30% G2 + 2SA formation. However, it cannot be concluded if the reduced activity is caused by the truncation of the enzyme or the FLAG Tag. A study on acceptor substrate specificity revealed a correlation between the enzyme length and activity [26]. The transfer efficiency was decreased up to 6-fold for truncated enzymatic forms (∆28-∆80) carrying an N-terminal Flag-Tag compared to the full-length enzyme. Legaigneur and co-workers [26] postulated that parts of the stem-region might participate in formation of the acceptor binding pocket. A recent crystal structure of human ST6Gal-1 reveals the possible binding mode of complex glycans [20] and shows important interactions of residues 108–122 with the glycan acceptor.

**Table 3:** **Analytic data of human FLAG-**Δ**108ST6Gal-I expressed under fed-batch conditions**

**Construct**	**Intensity of variant in ESMS [%]**	**MS analysis**	**Truncation**	**IgG1**		**Asialofetuin**
				**G2 + 1SA [%]**	**G2 + 2SA [%]**	**Fetuin [RFU/μ** **g]**
FLAG-Δ108ST6Gal-I*	85	DYKDDDDKLQKIWKN…	Δ108	14	0	33
	10	NYLS…	Δ114			
	5	YLS…	Δ115			

*The FLAG-Δ108ST6Gal-I construct was obtained from a 5-L fermentation.

It is also relevant to consider results of Malissard et al. [25] who have studied the expression of human ST6Gal-I in *P. pastoris* previously. The authors reported on the expression of a soluble ST6Gal-I with 3 U/L maximal activity in the culture supernatant. The current study extends the earlier work in presenting specific activities of purified enzymes on lactosamine as well as on non-sialylated protein-linked and released N-glycans. Enzyme activities are generally difficult to compare, but reported 3U/L exceed herein found 720 mU/L by 4-fold. However, one has to consider that the calculation of the data in this study is based on analysis of the purified protein without taking into consideration the potential loss of protein and activity caused by the downstream processing. Additionally, Malissard et al. measured the transfer reaction of CMP to LacNAc using a radiolabed sugar whereas in this study the transfer reaction rate was determined by the unlabeled and therefore less sensitive CMP-NANA. Beside the specific activities of purified enzymes, we show that N-terminal truncations are highly important for protein stability and activity and prove for the first time activity of a ∆108ST6Gal-I construct, while a deletion of 100 aa led to complete inactivation of human ST6Gal-I when transiently expressed in CHO cells [26].

## Conclusions

Precise analysis of degradation products, which were obtained during expression of N-terminally truncated human ST6Gal-I variants in *P.pastoris* KM71H, led to the identification of a minimal catalytic domain corresponding to Δ108ST6Gal-I. So far, it has been reported in literature that the deletion of more than 100 amino acids completely abolished enzyme activities. In this work we have demonstrated that N-terminal truncation of 108 amino acids maintained the activity. Additionally, expression of the N-terminal Flag-tagged Δ108ST6Gal-I clearly indicated an improved stability of the enzyme against proteolysis. FLAG-Δ108ST6Gal-I catalyzed the transfer of sialic acid to the galactosyl residues of a humanized antibody which verified the potential for *in-vitro* glycosylation of therapeutic proteins.

## Materials and methods

### Chemicals, Strains and Vectors

*E. coli* XL-1 Blue, *P.pastoris* KM71H and vector pPICZαB were purchased from Invitrogen (Austria). Asialofetuin, CMP-Neu5Ac, CMP-9F-Neu5Ac and cOmplete protease inhibitor tablets were obtained from Roche (Germany). Anti-α2,6-Sialytransferase© Rabbit IgG Antibody solution was purchased from IBL (Japan, #1898), monoclonal anti-polyHis from Sigma-Aldrich (Germany).

### General recombinant DNA techniques

Phusion DNA Polymerase (Finnzyme) and dNTP’s from MBI Fermentas (Germany) were used for PCR. The PCR was performed in a Gene Amp® PCR 2200 thermocycler (Applied Biosystems, USA). Digestion of DNA was performed with restriction endonucleases from New England Biolabs (USA). Wizard® Plus SV Minipreps DNA Purification System (Promega, Germany) was utilized to prepare plasmid DNA. PCR products and DNA fragments were purified by the Wizard® SV Gel and PCR Clean-up System (Promega, Germany).

### Construction and transformation of the recombinant plasmids

**Figure 4:**
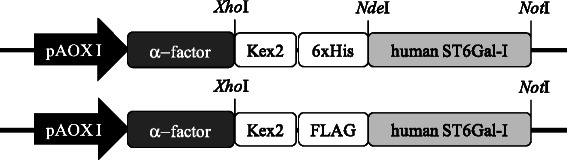
**Schematic representation of the pPICZαB-based** ***P.pastoris*** **expression vectors employed in this study.** pAOX1: alcohol oxidase 1 gene promoter; α-factor: coding region for the signal sequence of *S. cerevisae* α-mating factor; 6xHis: coding sequence for 6 histidines; Flag: coding sequence for the Flag-Tag; Kex2: coding sequence for Kex2 protease cleavage sites; human ST6Gal-I: coding sequence for the truncated variants of human ST6Gal-I.

The gene coding for the human ST6Gal-I lacking the transmembran anchor (Δ27ST6) was codon optimized for *P.pastoris* and synthesized by the GeneArt® Gene Synthesis Service (Life Technologies, Germany). The synthesized DNA fragment was digested with *Xho*I and *Not*I and cloned into pPICZαB behind the α-factor yielding pPICZαB_Δ27ST6 (Figure 4).

The DNA fragment included the *Xho*I restriction site, the *P.pastoris* Kex2 protease cleavage site, an ALE-6xHisTag coding peptide, the *Nde*I restriction site, the optimized gene, the stop codon and the *Not*I restriction site. The respective truncated versions Δ27ST6Gal-I, Δ48ST6Gal-I, Δ62ST6Gal-I, Δ89ST6Gal-I and Δ108ST6Gal-I of the gene encoding human ST6Gal-I were amplified by PCR from pPICZαB_Δ27ST6 using the primers listed in Additional file 4: Table S1. The following conditions were employed for PCR amplification (30 cycles): 98°C for 30 s, 98°C for 10 s, 62°C for 25 s, 72°C for 40 s and 72°C for 7 min with Phusion® DNA polymerase. The PCR mixtures were purified, digested with *Nde*I/*Not*I, ligated into pPICZαB_Δ27ST6 restricted with *Nde*I/*Not*I and transformed into *E.coli* XL-1 Blue. Identity of the cloned genes was confirmed by DNA sequencing.

Introduction of the N-terminal FLAG-Tag (Figure 4) was performed by PCR using plasmid pPICZαB_Δ108ST6 as template and the primer pair 5′-TATCTCTCGAGAAAA-GAGATTACAAGGATGACGACGATAAGTTGCAGAAGATTTGGAAGAACTACTTGC-CATGAACAAG-3′ for amplification. The reaction was performed with Phusion® DNA polymerase accordingly to the manufacturer’s instructions.

### Transformation and selection for multi-integration events

About 1 μg of each plasmid was linearized with *Sac*I and transformed into 80 μL of freshly prepared competent cells of KM71H by electroporation at 1500 V, 25 μF and 600 Ω using a Gene Pulser (BioRad). Transformed cells were plated out on agar plates supplemented with 100 μg/mL Zeocin and incubated at 30°C. Transformants were cultivated in microtiter plates and screened for multiple plasmid integration events on agar plates using Zeocin concentrations from 100 – 2,000 μg/mL. Selected clones were stored in MTP at −80°C.

### Expression of ST6Gal-I

#### Expression in Deep Well plates

Transformants were cultivated in deepwell plates (96-well format) in a Multitron II stackable incubation system (Infors, Bottmingen, Switzerland). Briefly, following a 12 h long incubation in 600 μL BYPD medium (1% yeast extract, 2% peptone, 2% glucose, 200 mM potassium phosphate buffer pH 7) at 320 rpm, 28°C and 80% air humidity, cells were induced by addition of BBM (1% glycerol, 1.34% yeast nitrogen base, 4×10^−5^% biotin, 1% (v/v) methanol, 200 mM potassium phosphate buffer pH 7). Following induction cycle was performed: 12 h 0.5% MeOH – 12 h 1% MeOH – 12 h 0.5% MeOH – 12 h 1% MeOH. After 48 h of induction, cells were harvested by centrifugation (4°C, 2000 rpm, and 20 min). Culture supernatants and cell pellets were used for analysis of activity and expression levels.

#### Expression in shake flasks

Transformants were used to inoculate a 1-L baffled shake flask containing 200 mL BMGY broth (1% yeast extract, 2% peptone, 1% glycerol, 4×10^−5^% biotin, 100 mM potassium phosphat buffer pH 6). The cultures were incubated at 28°C, 250 rpm for approximately 16–18 h until an OD_600_ of 2–6 was reached. The cells were harvested by centrifugation at 3,000 × g for 5 min and the cell pellet was resuspended in 1/10 of the original volume (20 mL). The culture was placed in a 100-mL baffled shake flask. Expression was induced by the addition of methanol to a final concentration of 0.5%. Samples were collected after 48, 72, 96 and 120 h of induction in the presence (+PI) or absence (−PI) protease inhibitor and stored at −20°C.

#### 5-L Fermentation

A similar work-up procedure, as described above, was used. In a first step, two liters of supernatant were centrifuged (15 min at 8,500 rpm), filtrated (0.2 μm), concentrated (to 150 mL, Pellicon 2 Mini Ultrafiltration Module, 10 kDa membrane, 0,1 m^2^ (Millipore)) and dialyzed against 20 mM potassium phosphate, pH 6.5 (buffer A) and concentrated. The dialysate was loaded onto an S-Sepharose FF column (5.0 × 5.1 cm), equilibrated with buffer A. After washing with 600 mL buffer A, the enzyme was eluted with a linear gradient of 0–200 mM NaCl within 200 mL. A second wash step with 200 mM NaCl (300 mL) was performed. Fractions (50 mL) were analyzed by SDS-PAGE.

### Purification of ST6Gal-I

#### From shake flasks

A two-step work-up procedure was used. In a first step, the culture supernatant was centrifuged (10 min at 3,000 rpm), filtrated (0.2 μm) and dialyzed against 20 mM potassium phosphate, pH 7.0. The dialysate was loaded onto a HisTrap HP FF 5-mL column (GE Healthcare, Germany) at a flow rate of 2 mL/min. After a washing step of 10 column volumes, the enzyme was eluted with a linear gradient of 0–500 mM imidazol within 10 column volumes. Fractions of 2-mL were collected and analyzed by SDS-PAGE. Sialyltransferase containing fractions were pooled and buffer was exchanged to 50 mM MES, pH 6.5.

#### From 5-L fermentation

A similar work-up procedure, as described above, was used. In a first step, two liters of supernatant were centrifuged (15 min at 8,500 rpm), filtrated (0.2 μm), concentrated (to 150 mL, Pellicon 2 Mini Ultrafiltration Module, 10 kDa membrane, 0,1 m2 (Millipore)) and dialyzed against 20 mM potassium phosphate, pH 6.5 (buffer A) and concentrated. The dialysate was loaded onto an S-Sepharose FF column (5.0 × 5.1 cm), equilibrated with buffer A. After washing with 600 mL buffer A, the enzyme was eluted with a linear gradient of 0–200 mM NaCl within 200 mL. A second wash step with 200 mM NaCl (300 mL) was performed. Fractions (50 mL) were analyzed by SDS-PAGE.

### SDS-PAGE and Immunoblotting

Analytical SDS gel electrophoresis was carried out using NuPAGE gels (4–12%, Invitrogen). Samples (36 μL) were diluted with 12 μL NuPAGE LDS sample buffer (Invitrogen) and incubated for 2 min at 85°C. Aliquots, typically containing 5 μg of protein were loaded on the gel. The gels were stained using SimplyBlue SafeStain (Invitrogen).

For Dot Blot analysis, 5 μL of the culture supernatant from deepwell cultivation were loaded onto a Biodyne® A nylon 6.6 membrane (pore size 0.45 μm, Pall Corporation, Germany) and tried for 1 h at 60°C. For Western Blot analyses, protein samples were separated by SDS-PAGE and blotted onto PVDF Transfer membranes (Amersham Hybond-P, GE Healthcare). Dot Blots and Western Blots were treated similarly for receptor detection: the membranes were washed twice with 20 mL TBS for 7 min at room temperature with shaking and blocked for 1 h at room temperature in TBS buffer (10 mM Tris HCl, pH 7.5 and 150 mM NaCl) containing 3% (w/v) BSA. After 2× washing with TBS-Tween/Triton buffer (Qiagen, 20 mM Tris HCl pH 7.5, 500 mM NaCl, 0.05% (v/v) Tween 20, 0.2% (v/v) Triton X-100) and 1× washing with TBS buffer (for each time at room temperature, 7 min with shaking), the membranes were incubated with the Anti-α2,6-Sialytransferase© Rabbit IgG Antibody solution (2 μg/mL dilution in blocking buffer of antibody) at room temperature for 1 h. The membranes were washed twice with TBS-Tween/Triton buffer and once with TBS buffer each time for 7 min at room temperature. Then the membrane was incubated with secondary antibody (1/5000), dilution in blocking buffer of antibody horseradish peroxidase (HRP conjugated) for 1 h at room temperature. The membranes were washed twice for 7 min with TBS-Tween/Triton buffer at room temperature followed twice for 7 min with TBS buffer and incubated with 7.5 mL of SuperSignal West Pico Substrate Working Solution for 3 min. Detection was performed using the G:Box F3 (Syngene).

### Mass spectrometry

The molecular masses of variants of human ST6Gal-I expressed in *P.pastoris* were analyzed by mass spectroscopy. Therefore, the deglycosylated forms of human ST6Gal-I were prepared and analyzed using Micromass Q-Tof Ultima and Synapt G2 HDMS devices (Waters UK) and the MassLynx V 4.1 software. For deglycosylation, samples were denatured and reduced; 45 μL denaturing buffer (6 M guanidine hydrochloride) and 13 μL TCEP (0.1 mM, diluted in denaturing buffer) were added to 100 μg sialyltransferase. An appropriate volume of ultrapure water was added to reach an overall concentration of about 4 M of guanidine hydrochloride. After incubation of the sample for 1 h at 37°C the buffer was changed using a Bio-SpinR 6 Tris column (BioRad), which was pre-equilibrated with ultrapure water. The whole sample was applied onto the column and eluted by centrifugation. To the resulting eluate, 5.5 μL of a 0.1 U/μL solution of PNGase F was added and the mixture was incubated at 37°C overnight. Afterwards the samples were adjusted to 30% ACN and 1% FA and analyzed by electrospray ionization mass spectrometry.

### N-terminal sequencing by Edman degradation

The N-terminal sequences of expressed variants of human ST6Gal-I were analyzed by Edman degradation using reagents and devices obtained from Life Technologies. Preparation of the samples was done as described in the instruction manual of the ProSorb Sample Preparation cartridges and the ProBlott Mini PK/10 membranes. For sequencing the Procise Protein Sequencing Platform was used.

### Activity assays

#### Fluorescence-based assay

Enzymatic activity was determined by measuring the transfer of fluorescence-labeled sialic acid to asialofetuin according to the literature [20]. Enzymatic activity was expressed as RFU/μg (relative fluorescence unit). 10,000 RFU/μg correspond to a specific activity of 0.084 U/mg.

#### HPLC-based assay

The reaction mixture consisted of 0.75 mM CMP-Neu5Ac, 10 mM lactosamine or 0.35 mM asialofetuin and 1 μM purified enzyme solution in 20 μL of 50 mM MES, pH 6.5 containing 0.1% Triton X-100. The enzymatic reaction was carried out at 37°C and 300 rpm. All assays were performed in duplicate. The enzymatic reaction was stopped after a certain time of incubation by quenching on ice and addition of 40 μL of ice-cold acetonitrile. The reaction mixture was centrifuged at 4°C, 13,000 rpm for 3 min to remove precipitated protein. After appropriate dilution, 10 μL were injected to HPLC analysis using a Chromolith® Performance RP-18 (100 × 4.6 mm; Merck Chemicals, Germany) column in reversed phase ion-pairing mode on an Agilent Technologies 1200 Series system. The column was equilibrated with 20 mM phosphate buffer, pH 6.8 containing 2 mM tetrabutylammonium at a flow rate of 2 mL/min. A temperature control unit maintained 30°C throughout the analysis. Samples were eluted with a linear gradient from 0–2% acetonitrile in 3 min followed by 2–25% acetonitrile in 7 min and detected by UV at 254 nm. The increase of CMP and the decrease of CMP-Neu5Ac were recorded. One unit (1 U) was defined as the amount of enzyme that could transfer 1 μmol of sialic acid per min to lactosamine under the conditions described above.

#### Hydrolase and Sialidase assay

The reaction mixture for determination of CMP-Neu5Ac hydrolase activity consisted of 0.75 mM of CMP-Neu5Ac and 1 μM of purified enzyme in 20 μL of 50 mM MES, pH 6.5 containing 0.1% Triton X-100. Reactions were allowed to proceed for 0.5, 10, 15, 30 and 60 min at 37°C and 300 rpm. The reaction products were analyzed by HPLC as described above.

The reaction mixture for determination of α2,6-sialidase activity consisted of 1.5 mM of 6′-sialyllactosamine, 0 or 1.0 mM CMP and 1 μM of purified enzyme in 20 μL of 50 mM MES, pH 6.5 containing 0.1% Triton X-100. Reactions were allowed to proceed for up to 22 h at 37°C and 300 rpm. The reaction products were analyzed by HPAE chromatography on a Dionex BioLC system equipped with a CarboPac® PA200 column (3 × 250 mm; Thermo Fisher Scientific Inc., Dionex) and a CarboPac® guard column. 25 μL of sample were injected and eluted using an isocratic concentration of 100 mM NaOH with 40 mM sodium acetate and a flow rate of 0.5 mL/min at 30°C. An ED50 electrochemical detector with a carbohydrate certified gold working electrode was used for pulsed amperometric detection (PAD) in the carbohydrate waveform (as recommended from the supplier). Increase of sialic acid and decrease of 6′-sialyllactosamine was recorded.

### Sialylation of monoclonal antibody

A highly galactosylated humanized monoclonal antibody IgG1 was used in sialylation experiments. The reaction mixture contained IgG1 (300 μg in 54 μL 35 mM sodium acetate/Tris buffer pH 7.0), the donor substrate CMP-Neu5Ac (150 μg in 50 μL water) and sialyltransferase (30 μg in 26 μL 20 mM potassium phosphate, 0.1 M NaCl, pH 6.5). The samples were incubated at 37°C for a defined time. To stop the reaction 100 μL denaturing buffer (6 M guanidine hydrochloride) and 30 μL TCEP (0.1 mM, diluted in denaturing buffer) were added to the samples and the samples were incubated at 37°C for 1 h. The samples were buffered in electrospray-medium (20% ACN, 1% FA) using pre-equilibrated illustraTM Nap5-Columns (GE-Healthcare). Samples were analyzed by electrospray ionization mass spectrometry and the content of G2 + 0SA, G2 + 1SA and G2 + 2SA N-glycans was determined. A Micromass Q-Tof Ultima and a Synapt G2 HDMS device (Waters UK) and the MassLynx V 4.1 software were used.

## Additional files

Additional file 1: Figure S1. SDS-PAGE of purified ST6Gal-I expressed in *P.pastoris* KM71H with protease inhibitors. *Lane 1*: ∆62ST6Gal-I; *lane 2*: ∆48ST6Gal-I; *lane 3*: ∆89ST6Gal-I; *lane 4*: Novex Sharp Protein Standard. Additional file 2: Figure S2. Sialylation of Fc glycan. Additional file 3: Figure S3. Expression of ∆108ST6Gal-I without Tag **(A)** and with N-terminal HisTag **(B)** in the presence of protease inhibitor. Expression analysis by Western Blot/ anti-ST6Gal-I and SDS-PAGE. Additional file 4: Table S1. Primer sequences for truncation of human ST6Gal-I.

## Abbreviations

RFU: Relative fluorescence unit
BYPD: Buffered yeast extract peptone dextrose
BMGY: Buffered Glycerol-complex Medium
TBS: Tris buffered saline
FA: Formic acid
HPAE chromatography: High-Performance Anion-Exchange Chromatography
TCEP: Tris(2-CarboxyEthyl)Phosphine
