# Supplementary material for: High-quality production of human α-2,6-sialyltransferase in Pichia pastoris requires control over N-terminal truncations by host-inherent protease activities
Source: Microb Cell Fact. 2014 Sep 11;13:138. doi: 10.1186/s12934-014-0138-8 (PMC4172862; doi:10.1186/s12934-014-0138-8)
Supplement: Additional file 1: Figure S1. — SDS-PAGE of purified ST6Gal-I expressed in P.pastoris KM71H with protease inhibitors. Lane 1: ∆62ST6Gal-I; lane 2: ∆48ST6Gal-I; lane 3: ∆89ST6Gal-I; lane 4: Novex Sharp Protein Standard. [file 12934_2014_138_MOESM1_ESM.docx]

**Additional file 1: Figure S1.**SDS-PAGE of purified ST6Gal-I expressed in *P.pastoris* KM71H with protease inhibitors. *Lane 1*: Δ62ST6Gal-I; *lane 2*: Δ48ST6Gal-I; *lane 3*: Δ89ST6Gal-I; *lane 4*: Novex Sharp Protein Standard.
