# Supplementary material for: High-quality production of human α-2,6-sialyltransferase in Pichia pastoris requires control over N-terminal truncations by host-inherent protease activities
Source: Microb Cell Fact. 2014 Sep 11;13:138. doi: 10.1186/s12934-014-0138-8 (PMC4172862; doi:10.1186/s12934-014-0138-8)
Supplement: Additional file 2: Figure S2. — Sialylation of Fc glycan. [file 12934_2014_138_MOESM2_ESM.docx]

**

**Additional file 2: Figure S2.** Sialylation of Fc glycan.

Galacatose; Sialic acid
