# Supplementary material for: High-quality production of human α-2,6-sialyltransferase in Pichia pastoris requires control over N-terminal truncations by host-inherent protease activities
Source: Microb Cell Fact. 2014 Sep 11;13:138. doi: 10.1186/s12934-014-0138-8 (PMC4172862; doi:10.1186/s12934-014-0138-8)
Supplement: Additional file 3: Figure S3. — Expression of ∆108ST6Gal-I without Tag (A) and with N-terminal HisTag (B) in the presence of protease inhibitor. Expression analysis by Western Blot/ anti-ST6Gal-I and SDS-PAGE. [file 12934_2014_138_MOESM3_ESM.docx]

**

**Additional file 3: Figure S3.** Expression of Δ108ST6Gal-I without Tag (A) and with N-terminal HisTag (B)

in the presence of protease inhibitor. Expression analysis by Western Blot/ anti-ST6Gal-I and SDS-PAGE.
