# Supplementary material for: High-quality production of human α-2,6-sialyltransferase in Pichia pastoris requires control over N-terminal truncations by host-inherent protease activities
Source: Microb Cell Fact. 2014 Sep 11;13:138. doi: 10.1186/s12934-014-0138-8 (PMC4172862; doi:10.1186/s12934-014-0138-8)
Supplement: Additional file 4: Table S1. — Primer sequences for truncation of human ST6Gal-I. [file 12934_2014_138_MOESM4_ESM.docx]

**Additional file 4: Table S1 Primer sequences for truncation of human ST6Gal-I.**

| **Primer name** | **Nucleotide sequences** |
| --- | --- |
| ∆48_hST6Gal-I.FW | 5’-CTGGAGATACT CATATG AAA TCC TTA GGC AAG TTA GCT ATG GGG T-3’ |
| ∆62_hST6Gal-I.FW | 5’-CTGGAGATACT CATATG GTT TCC TCA TCC TCC ACT CAA GAC C-3’ |
| ∆89_hST6Gal-I.FW | 5’-CTGGAGATACT CATATG GAA GCT TCT TTC CAG GTT TGG AAC AAG GAC -3’ |
| ∆108_hST6Gal-I.FW | 5’- ATCATCATATG TTG CAG AAG ATT TGG AAG AAC TAC TTG TCC ATG -3’ |
| hST6Gal-I.REV | 5’-CTGGAGATACT GCGGCCGCTCAACAGTG -3’ |
